# PS-09 - INVESTIGATION INTO POSSIBLE INCREASED SUSCEPTIBILITY TO THE COVID-19 BA.3.2 VARIANT IN CHILDREN

**DOI:** 10.1093/pubmed/fdag046.058

**Published:** 2026-07-09

**Authors:** Dr Kirstin Leslie, Mr Robert Norrie, Mr Allan McLeod, Dr Josie Evans, Professor Matthew Holden, Mr Sharif Shaaban, Professor Chris Robertson, Dr Kimberly Marsh

**Affiliations:** Public Health Scotland; Public Health Scotland; Public Health Scotland; Public Health Scotland; Public Health Scotland; University of St Andrews; Public Health Scotland; Public Health Scotland; University of Strathclyde; Public Health Scotland

## Abstract

**Background:**

During the 2025/26 winter season, Scotland experienced a small COVID-19 wave linked to the emergence of BA.3.2, characterised by an unusually young age profile. BA.3.2, a highly divergent Omicron subvariant, has reduced antibody neutralisation and potential for immune escape. The wave occurred alongside an early H3N2-dominant influenza season, which typically causes more severe illness in children. This, coupled with widespread use of multiplex testing for influenza, RSV and COVID-19, may have led to incidental detection of BA.3.2 in younger ages.

**Objectives:**

Using Scotland’s comprehensive COVID-19 surveillance and sequencing data to determine whether the age profile observed is due to changed susceptibility to BA.3.2 or an artefact of testing patterns during a period of increased influenza transmission in children.

**Methods:**

Time series analysis of BA.3.2 by age was undertaken. Overall PCR test positivity and COVID-19 admissions were used to assess epidemiological trends as BA.3.2 emerged. These were considered against overall testing numbers to explore potential differential ascertainment in children.

**Results:**

BA.3.2 emerged earlier and accounted for a higher proportion of sequenced cases in children aged 1-14 (53.6%) compared to adults (22.7%) over the period. Increased BA.3.2 detections in children continued after the H3N2-influenza peak and in January exceeded 75%. During this time, overall COVID-19 test positivity breached 10% in children but not in adults. Admissions in children aged 1-4 were higher in early 2026 than any point in the previous 3 seasons, accounting for 17% (126/ 740) of total admissions but just 6.6% (2,159/ 54,218) of PCR tests. Test numbers by age were in alignment with previous seasons.

**Conclusions:**

As COVID-19 testing patterns remained consistent, the wave in children could not be attributed to higher testing for H3N2-influenza. Increased susceptibility due to changed immune properties of BA.3.2 may have caused higher-than-expected COVID-19 hospitalisations in children, particularly those aged 1-4.

